# Supplementary material for: Quantitative earthquake-like statistical properties of the flow of soft materials below yield stress
Source: Nat Commun. 2020 Jan 7;11:9. doi: 10.1038/s41467-019-13790-2 (PMC6946698; doi:10.1038/s41467-019-13790-2)
Supplement: Supplementary file 1 — Supplementary Information [file 41467_2019_13790_MOESM1_ESM.pdf]

Supplementary Information

Quantitative earthquake-like statistical properties of the flow of soft materials below yield stress

Bera et al.

Supplementary Figures

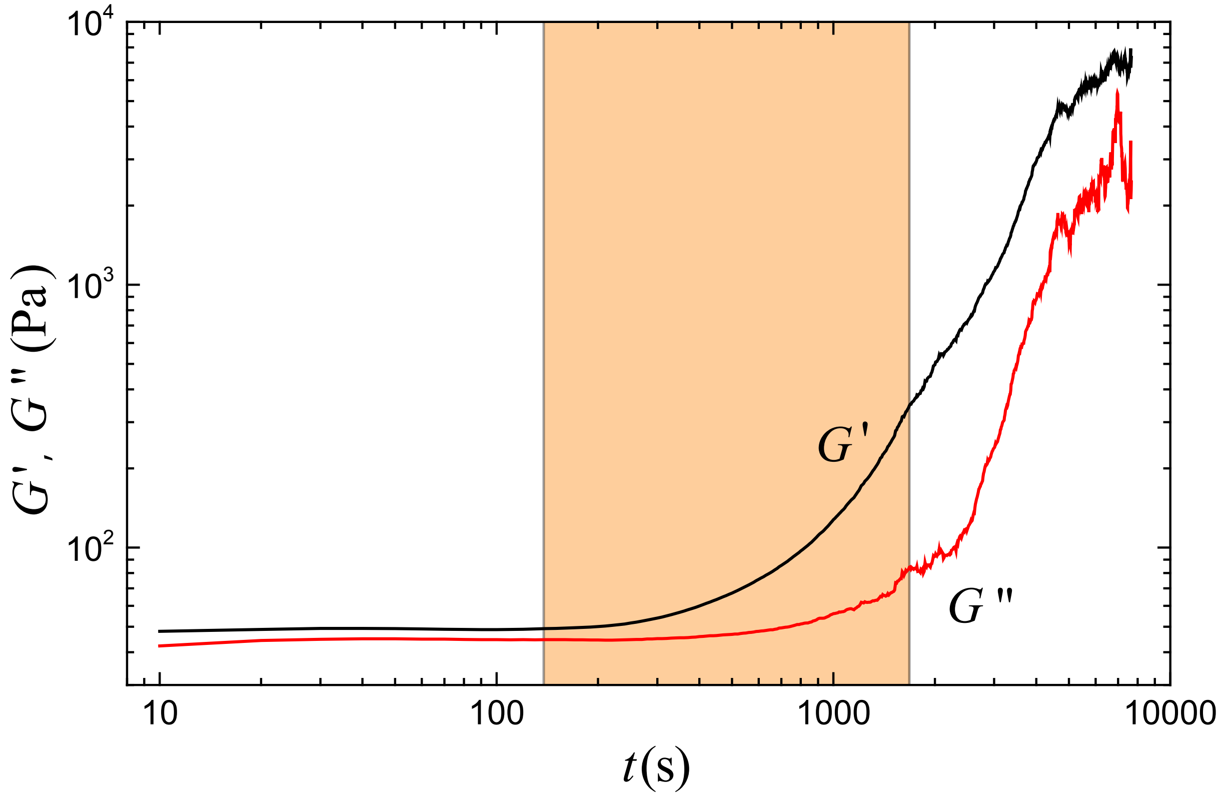

Supplementary Figure 1: **Oscillatory shear stress test on top of the constant stress.** Increment in storage modulus (G') and loss modulus (G'') with time, measured with DC (10 Pa) + AC (5 Pa, f = 1 Hz) applied stress, with 39 wt% CTAT-Water sample at 30° C with $d = 10$ μm. The shaded region indicates the time interval of the burst.

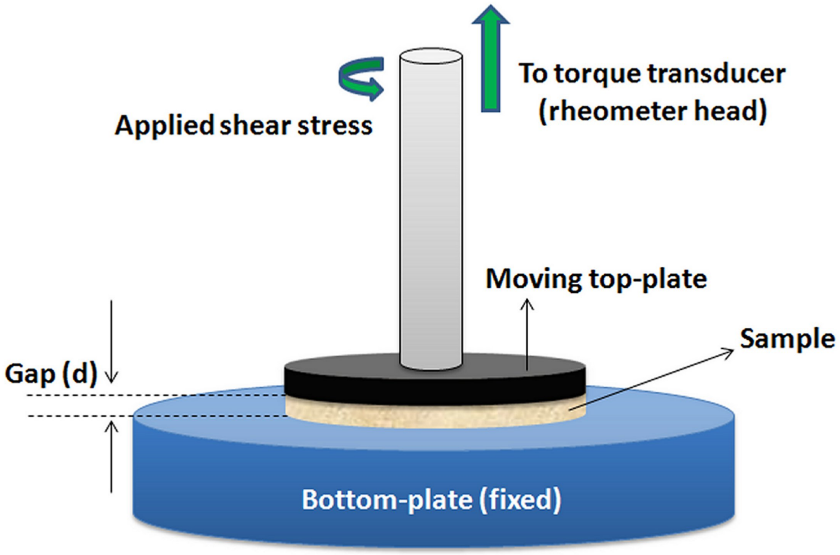

Supplementary Figure 2: **Experimental set-up.**

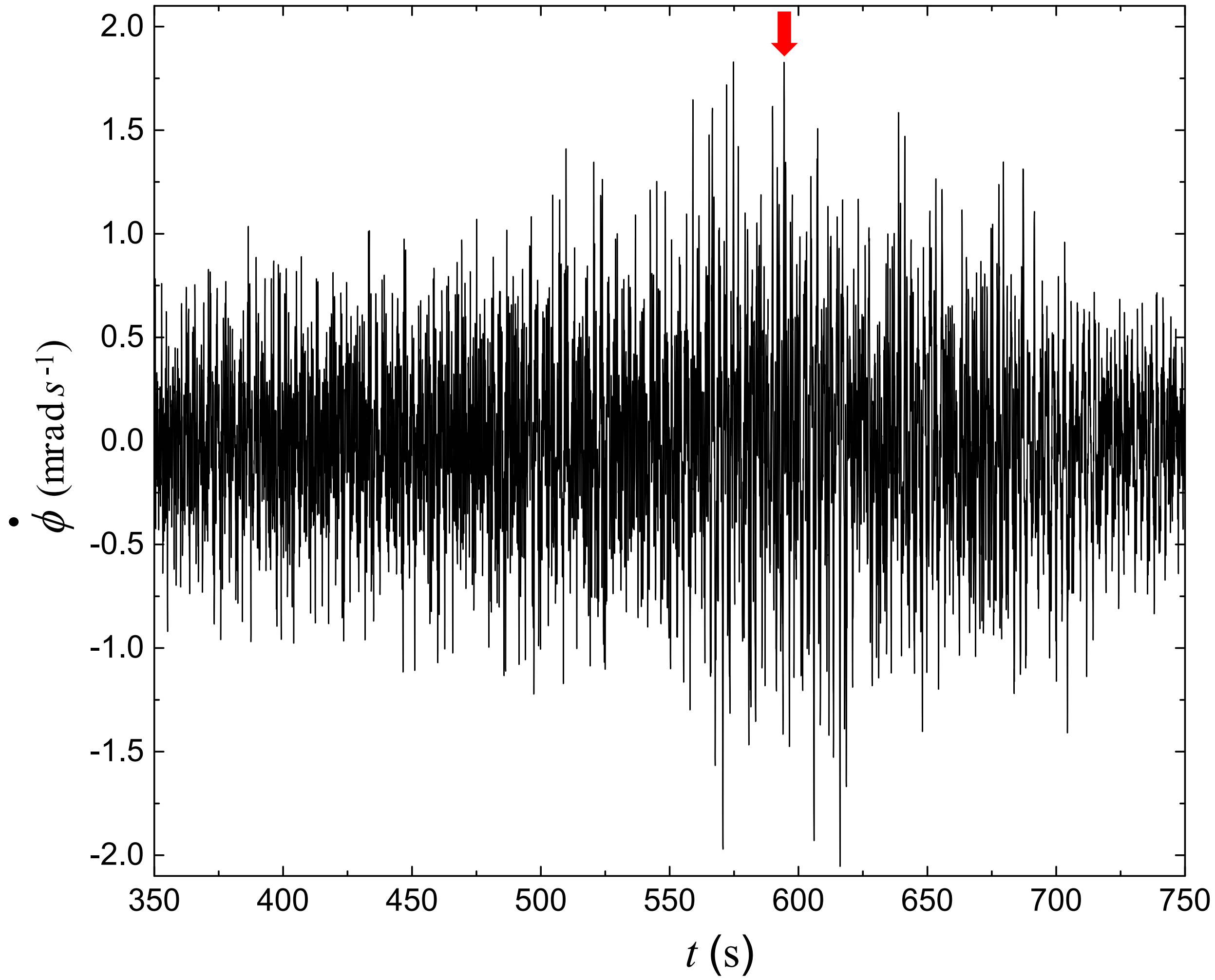

Supplementary Figure 3: **The burst in expanded version.** Expanded version of the data for $\sigma = 2$ Pa ($d = 10$ μm, main text Fig. 1b) in the time window 350 s to 750 s. The main shock at τ_s is marked by the down arrow.

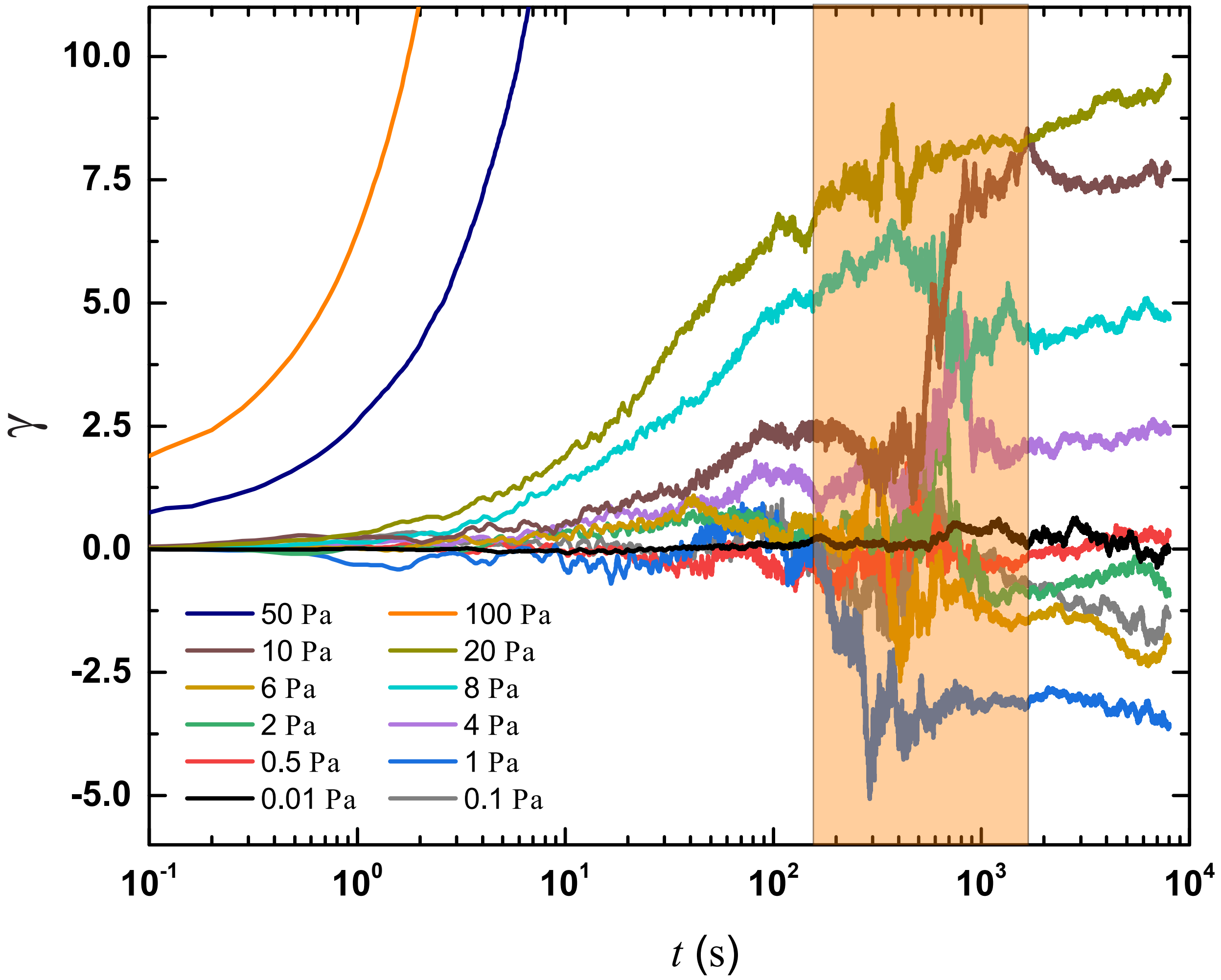

Supplementary Figure 4: **Net deformation of the material in time.** Cumulative strain (γ) vs t for different stress values are plotted. The shaded region indicates the time interval of the burst showing large jumps in γ. Sample: 39 wt% CTAT-Water at 30° C ($d = 10$ μm).

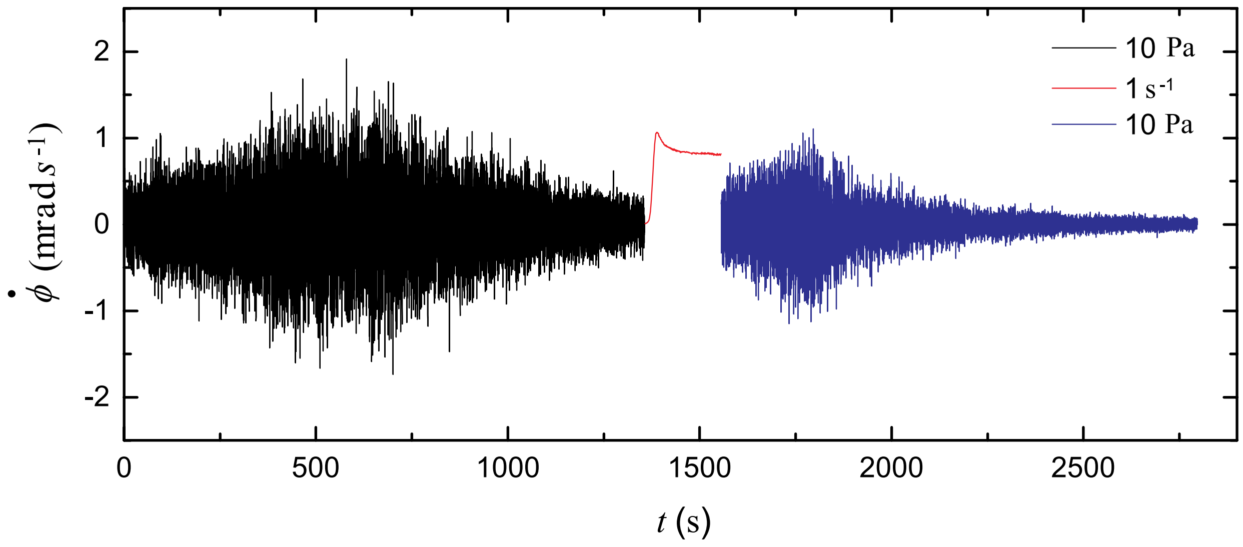

Supplementary Figure 5: **Burst return study with CTAT.** $\dot{\phi}$ vs t for 39 wt% CTAT-Water sample at 30° C ($d = 10$ μm) is plotted for the applied stress 10 Pa (black), then for the applied preshear 1 s^{-1} with duration 200 sec (red) and then for the applied stress again 10 Pa (blue).

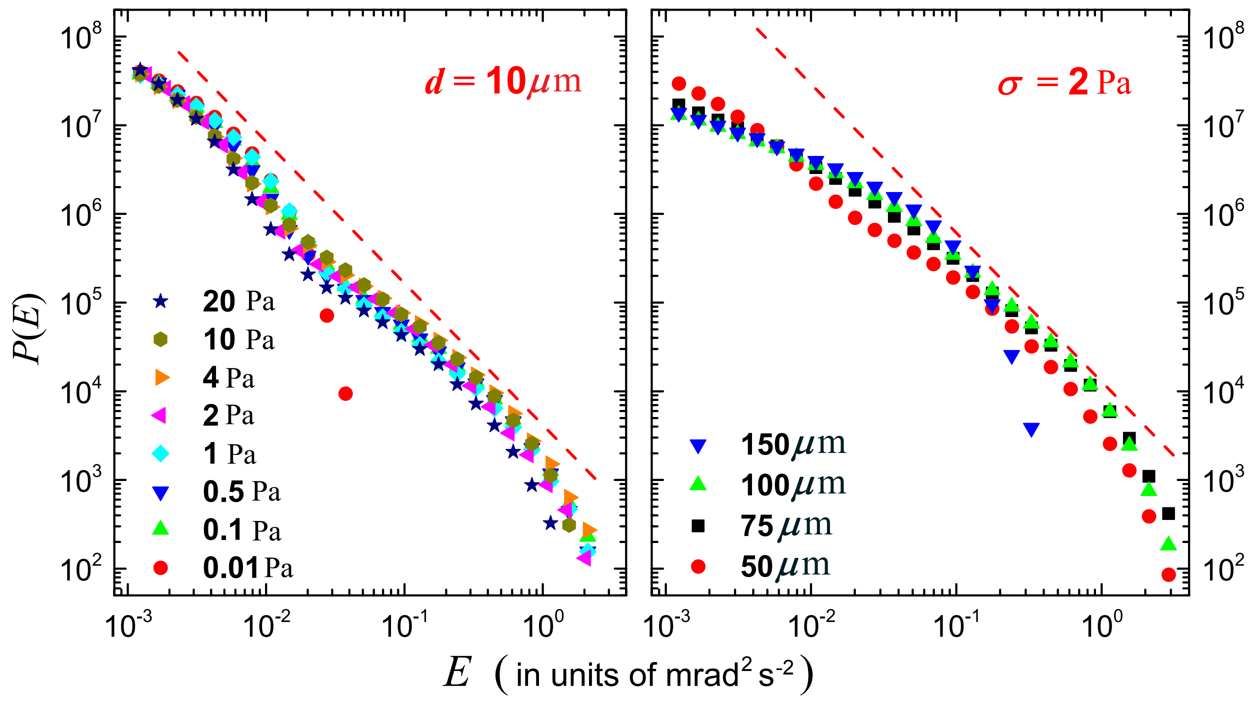

Supplementary Figure 6: **Gutenberg-Richter law for other bursts in CTAT nematic.** $P(E)$ vs E for different σ having $d = 10$ μm (left panel) and for different d having $\sigma = 2$ Pa (right panel) is shown for 39 wt% CTAT-Water sample at 30° C. The dashed lines indicate power-law decay with the slope $\epsilon = 1.6 \pm 0.1$ as in main text Fig. 2a.

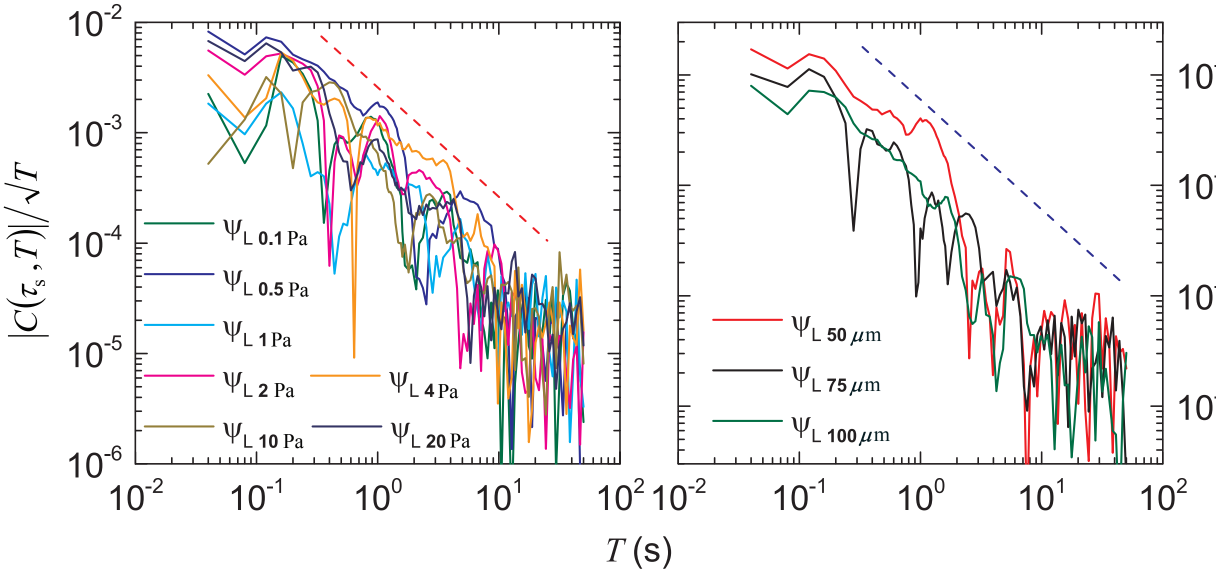

Supplementary Figure 7: **Omori law for other bursts in main text Fig. 1b,c.** Normalized wavelet coefficient vs T (using ψ^L as the analyzing wavelet for points on the left side of the burst) for different σ having $d = 10$ μm are plotted in the left panel, and for different d having $\sigma = 2$ Pa are plotted in the right panel. Dashed lines indicate power-law decay with exponent $\alpha = 1$.

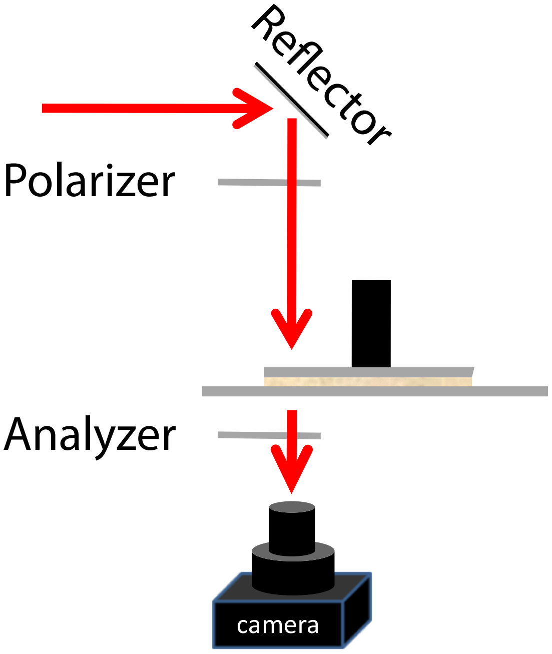

Supplementary Figure 8: **Home-built rheo-POM set-up for in situ microscopy.** The bottom glass plate is fixed and the upper glass plate is coupled to Rheometer. Polarizer axis is along the vorticity direction and analyzer axis is along the velocity direction.

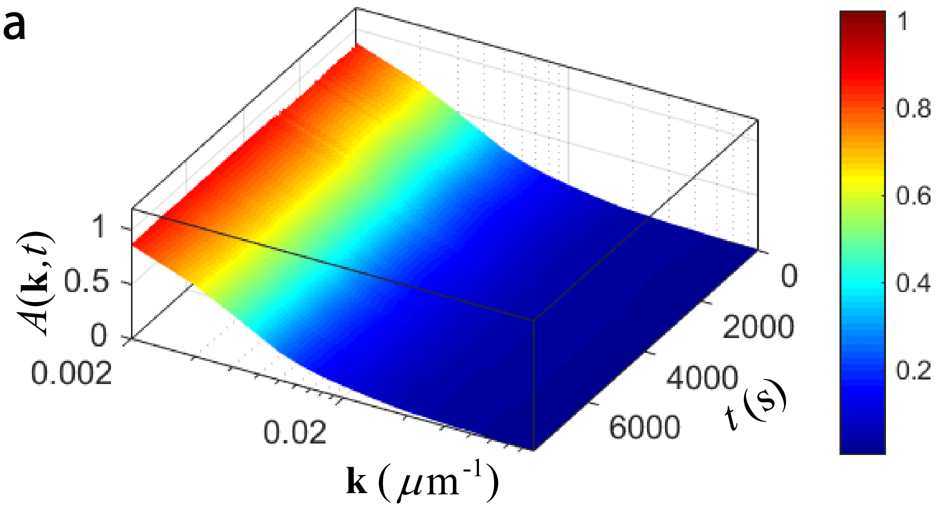

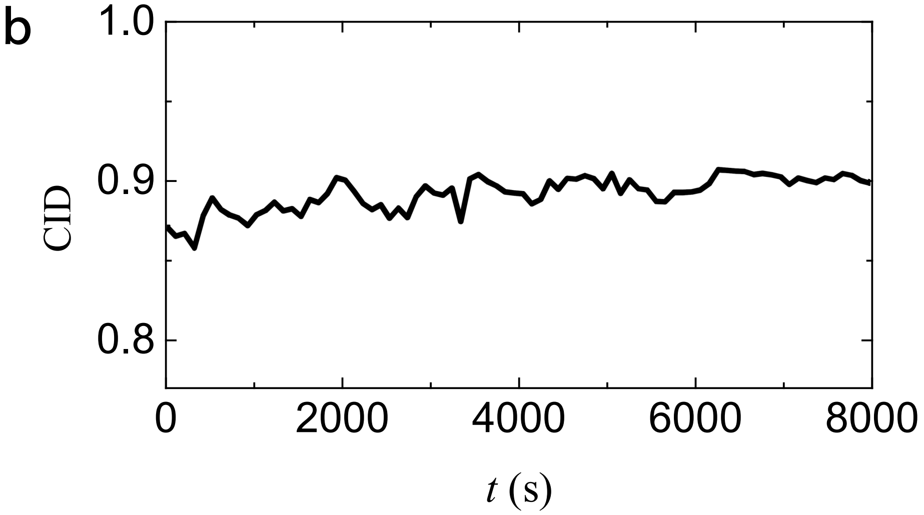

Supplementary Figure 9: **Microscopy of domain reorganization in CTAT nematic with no shear condition.** **a** $A(\mathbf{k}, t)$ vs $\mathbf{k}$, t and **b** CID vs t plots with $\sigma = 0$.

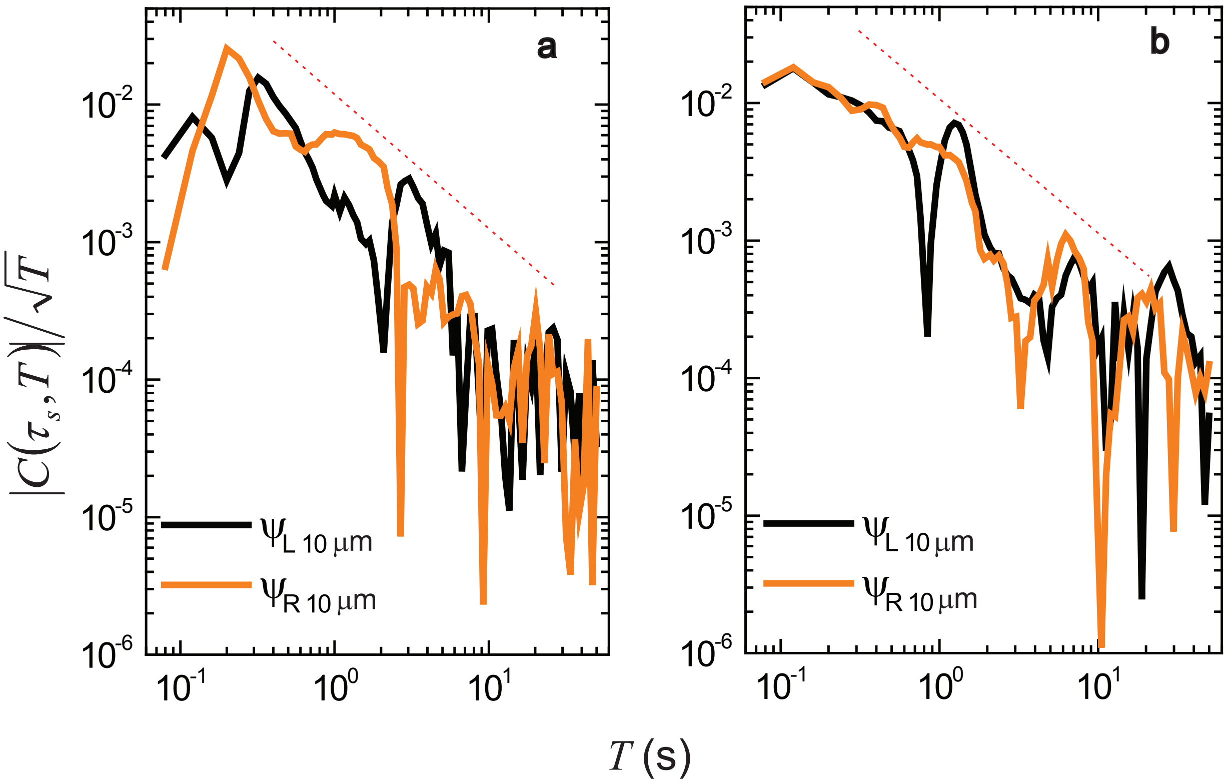

Supplementary Figure 10: **Omori law for other bursts marked B and C in the main text Fig. 5a.** Normalized wavelet coefficients near the singularities, marked B and C in main text Fig. 5a for the laponite sample (d = 10 μm) are shown in **a**,**b** respectively. Dashed lines indicate power-law decay with exponent $\alpha = 1$.

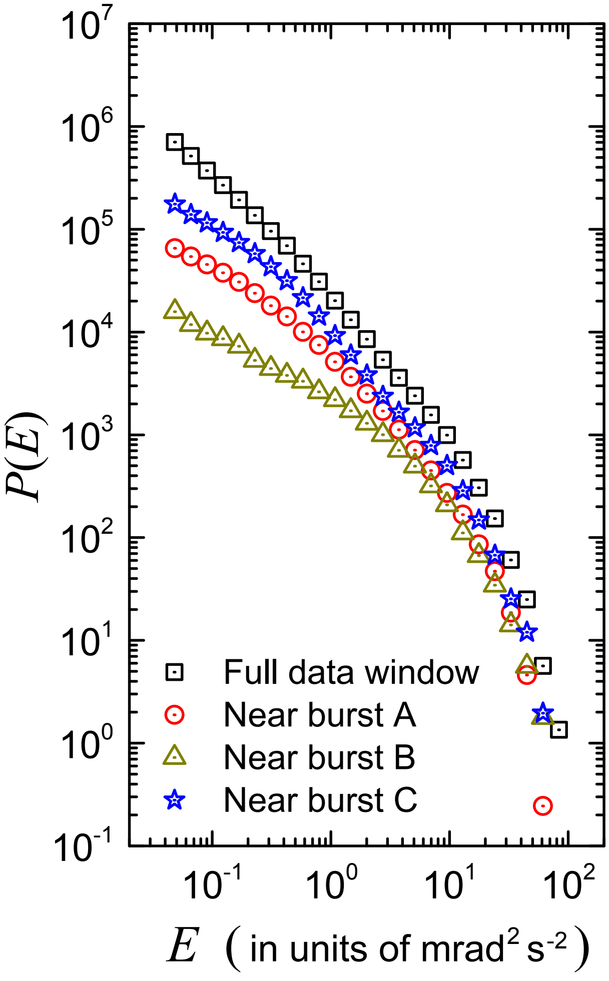

Supplementary Figure 11: **Gutenberg-Richter law for other bursts marked B and C in the main text Fig. 5a.** $P(E)$ vs E plot of laponite $d = 10$ μm data (main text Fig. 5a), considering the full time window and around the bursts A, B, C separately.

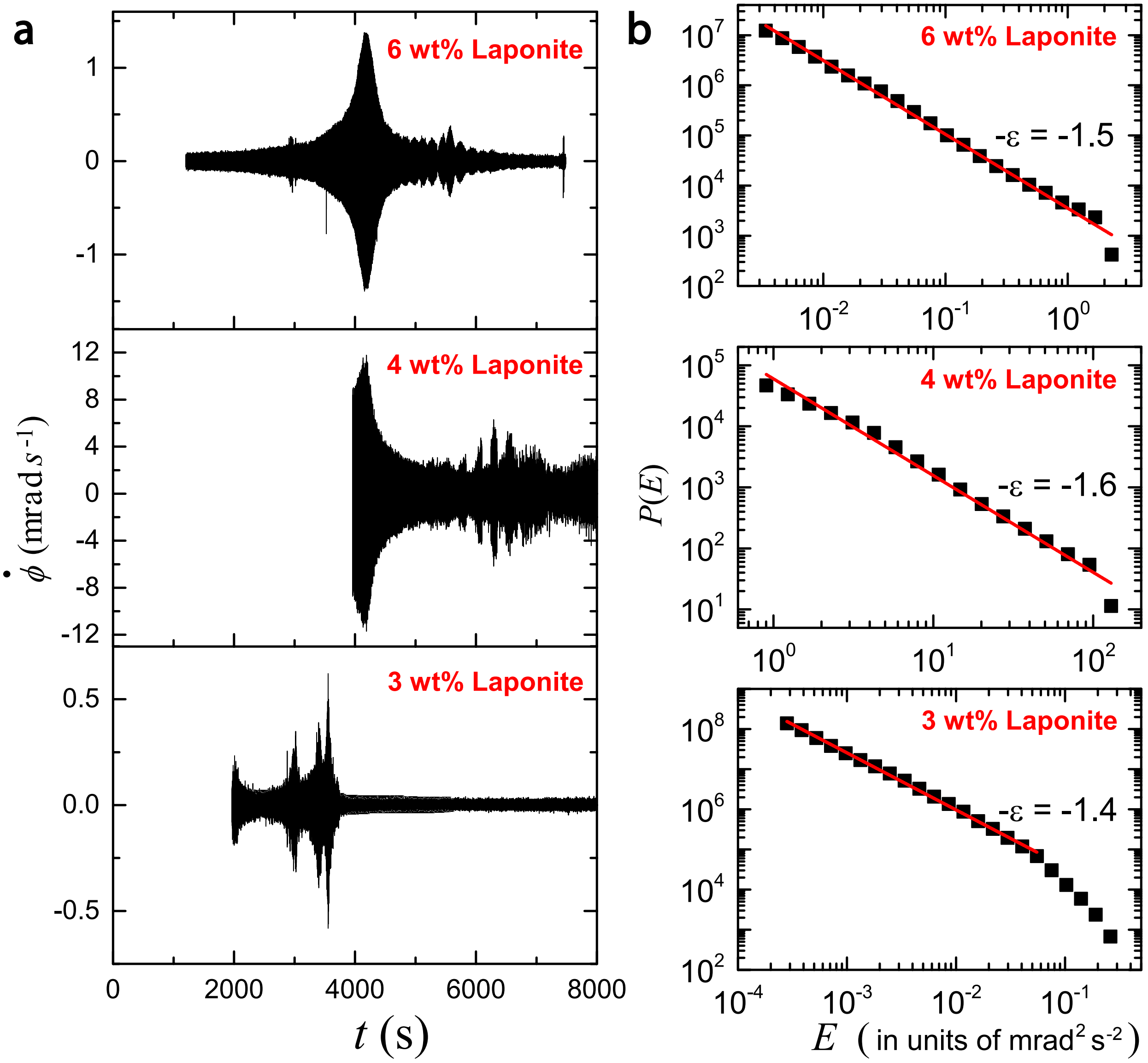

Supplementary Figure 12: **Bursts with other laponite sample compositions.** **a** $\dot{\phi}$ vs t plot for three different laponite sample compositions. **b** Corresponding $P(E)$ vs E plot.

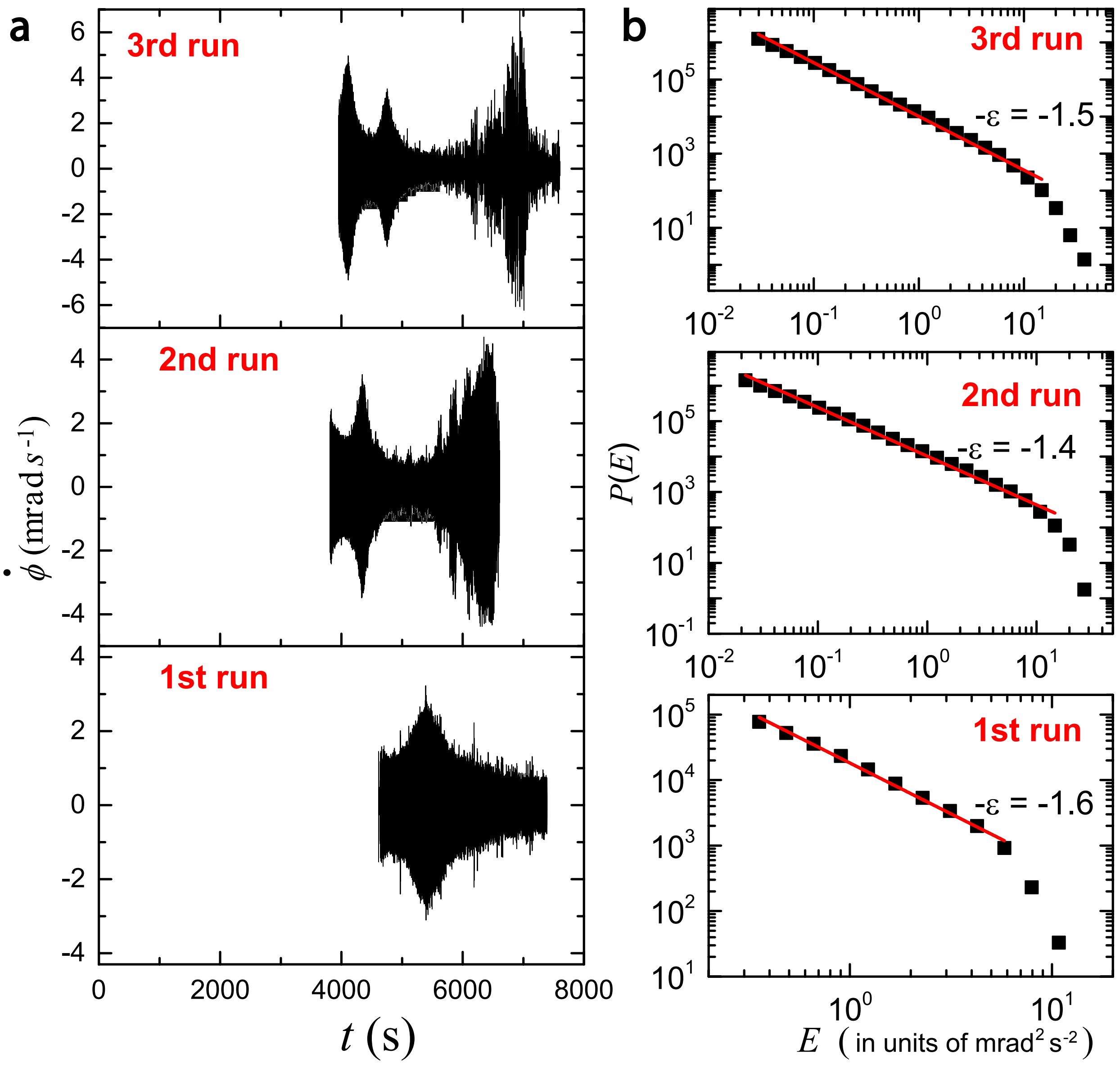

Supplementary Figure 13: **Burst return study with laponite. a** $\dot{\phi}$ vs t plot for three successive measurement runs having the same preshear protocol on the same loaded sample with 5 wt% laponite ($d = 40$ μm and $\sigma = 2$ Pa). **b** Corresponding $P(E)$ vs E plot.

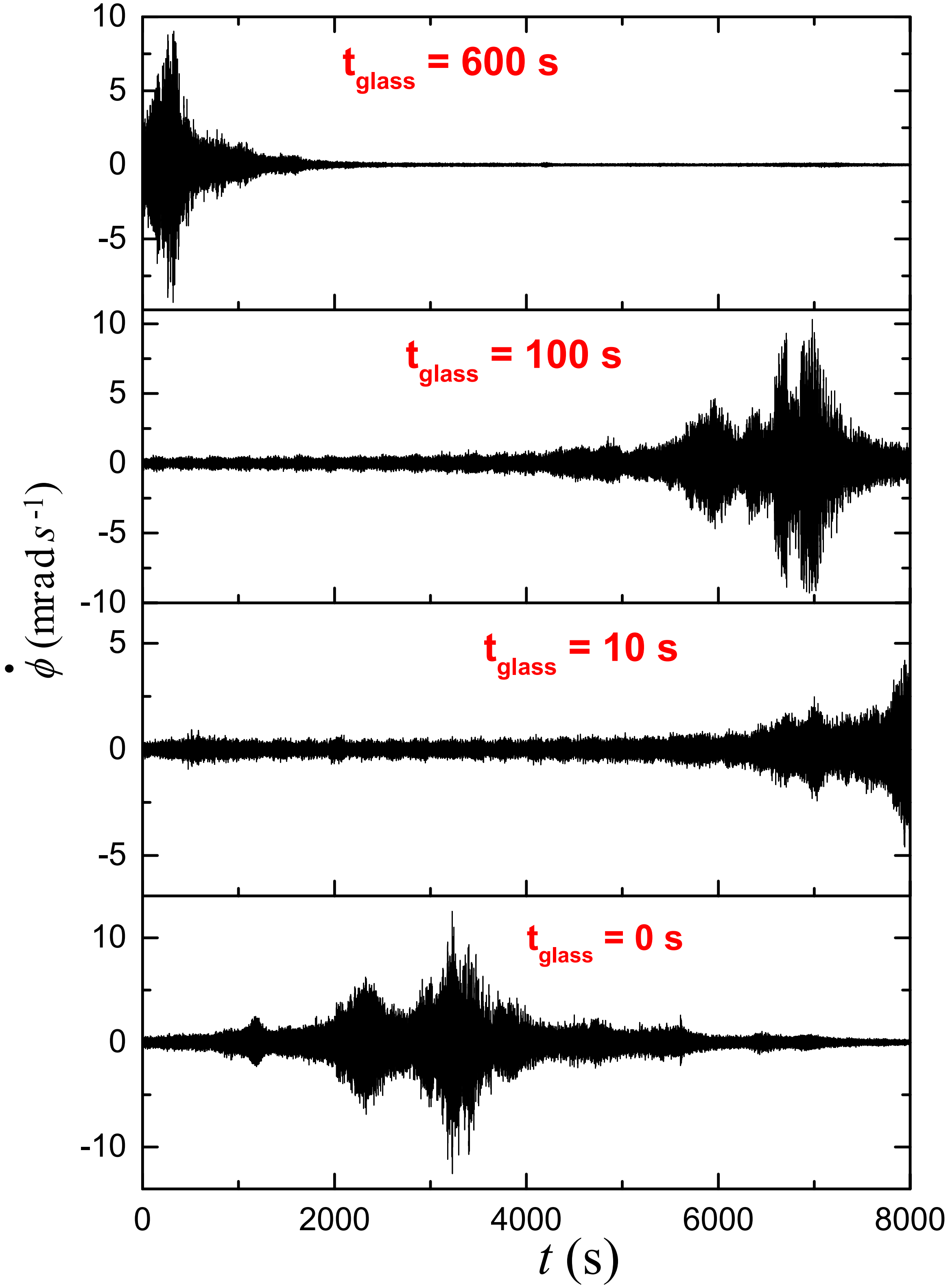

Supplementary Figure 14: **Bursts with different laponite sample aging.** $\dot{\phi}$ vs t plot for four successive measurements with different waiting times after preshear (t_{glass}) to allow the system to explore the glassy state after rejuvenation (5 wt% laponite having $d = 10$ μm and $\sigma = 2$ Pa).
