# Supplementary material for: Quantitative earthquake-like statistical properties of the flow of soft materials below yield stress
Source: Nat Commun. 2020 Jan 7;11:9. doi: 10.1038/s41467-019-13790-2 (PMC6946698; doi:10.1038/s41467-019-13790-2)
Supplement: Supplementary file 3 — Description of Additional Supplementary Files [file 41467_2019_13790_MOESM3_ESM.pdf]

# Description of Additional Supplementary Files

**Supplementary Movie1:**
100 fps Movie; constructed using POM images obtained during the rheo-POM measurement of 39 wt% CTAT + Water at 30$^{\circ}$ C with d = 150 µm and σ = 2 Pa (Left panel). Right panel is the corresponding binary version for better visibility.

**Supplementary Movie2:**
100 fps Movie; with no shear condition.
